# Prevalence and Patterns of Dyslipidemia and Associated Cardiometabolic Comorbidities Among Children With Overweight and Obesity Aged 5-16 Years Attending a Tertiary Care Center in Chennai, South India

**DOI:** 10.7759/cureus.114152

**Published:** 2026-08-07

**Authors:** Renuka Chandrasekaran, Harsha Vardhini Nagavel Sekaran, Shyamala J

**Affiliations:** 1 Pediatrics, Apollo Hospitals, Chennai, IND; 2 Pediatrics, SRM Medical College Hospital and Research Centre, Chengalpattu, IND

**Keywords:** childhood hypertension, dyslipidemia, fatty liver, lipid profile, metabolic syndrome, obesity, overweight, pcos, pediatric diabetes

## Abstract

Background: Childhood obesity has emerged as a major public health concern worldwide and is associated with several cardiometabolic complications, including dyslipidemia, hypertension, metabolic dysfunction-associated steatotic liver disease (MASLD), and polyendocrine metabolic ovarian syndrome (PMOS, previously termed polycystic ovary syndrome (PCOS)). Data regarding the prevalence and patterns of dyslipidemia among Indian children with overweight and obesity remain limited. Early identification of these abnormalities may help reduce future cardiovascular risk.

Methods: This prospective observational study was conducted among 100 children with overweight or obesity aged 5-16 years who attended the pediatric outpatient department of a tertiary care center in South India between April 2018 and May 2019. Anthropometric measurements, blood pressure assessment, fasting lipid profile, liver function tests, and abdominal ultrasonography were performed. Postmenarchal girls were additionally evaluated for PMOS. Dyslipidemia, hypertension, and MASLD were identified according to standard criteria. Statistical analyses were performed to determine the associations between obesity-related comorbidities and clinical variables.

Results: Among the 100 participants, 53 (53%) were female, and the mean age was 11.6 ± 2.56 years. Dyslipidemia was identified in 84 children (84%). The most common lipid abnormality was low high-density lipoprotein cholesterol (HDL-C) (57, 57%), followed by elevated triglycerides (44, 44%), elevated non-high-density lipoprotein cholesterol (non-HDL-C) (14, 14%), elevated total cholesterol (8, 8%), and elevated low-density lipoprotein cholesterol (LDL-C) (2, 2%). MASLD was detected in 32 children (32%), while hypertension was present in seven children (7%). Dyslipidemia was significantly more common among females than among males (94.3% vs. 72.3%; χ² = 8.97, p = 0.003). Hypertension was significantly more prevalent among children with obesity than among children with overweight (20% vs. 3.8%; OR = 6.42, p = 0.028). Among the 15 postmenarchal girls screened, four (26.7%) were found to have PMOS.

Conclusions: A high prevalence of dyslipidemia was observed among children with overweight and obesity, with low HDL-C and hypertriglyceridemia constituting the predominant pattern. The substantial burden of dyslipidemia, MASLD, hypertension, and PMOS highlights the need for early metabolic screening and targeted lifestyle interventions in children with excess adiposity.

## Introduction

Childhood obesity has become one of the most significant public health challenges of the twenty-first century. The increasing prevalence of overweight and obesity among children and adolescents has resulted in a parallel rise in obesity-related metabolic disorders, many of which were previously considered diseases of adulthood. Current evidence indicates that excess adiposity during childhood contributes substantially to future cardiovascular morbidity and mortality through the early development of metabolic risk factors and atherosclerotic changes [1-4].

India has undergone a substantial epidemiological transition over the past two decades, characterized by rapid urbanization, reduced physical activity, and changes in dietary habits. Consequently, the prevalence of overweight and obesity among Indian children has increased steadily across all socioeconomic groups [5,6]. This trend is particularly concerning because obesity acquired during childhood frequently persists into adulthood and is associated with long-term cardiometabolic complications.

Dyslipidemia is among the most common metabolic abnormalities observed in children with overweight and obesity, yet it remains underrecognized in routine pediatric practice despite its long-term cardiovascular implications [7,8]. It is characterized by elevated levels of total cholesterol (TC), low-density lipoprotein cholesterol (LDL-C), non-high-density lipoprotein cholesterol (non-HDL-C), and triglycerides (TG), as well as reduced levels of high-density lipoprotein cholesterol (HDL-C). The relationship between dyslipidemia and cardiovascular disease is well established. Atherosclerotic changes have been shown to begin during childhood, and lipid abnormalities during early life contribute to the accelerated progression of vascular disease [2,9-12]. Importantly, these early changes may be reversible with timely identification and intervention.

Beyond dyslipidemia, childhood obesity is associated with several additional comorbidities that further increase cardiometabolic risk. Children with obesity are at an increased risk of developing hypertension, which further amplifies cardiovascular risk [13]. Multiple mechanisms have been implicated in obesity-associated hypertension, including insulin resistance, activation of the sympathetic nervous system, altered vascular function, and increased sodium retention [14]. Furthermore, the coexistence of dyslipidemia and hypertension substantially increases the risk of future cardiovascular disease [2]. Similarly, metabolic dysfunction-associated steatotic liver disease (MASLD), formerly known as nonalcoholic fatty liver disease (NAFLD), has emerged as an important hepatic complication of pediatric obesity [15]. MASLD represents the hepatic manifestation of metabolic dysfunction and is increasingly recognized among children with excess body weight [16]. Because its early stages are often asymptomatic, screening is particularly important in high-risk populations [17]. Among adolescent girls, obesity has also been associated with polycystic ovary syndrome (PCOS), a common endocrine disorder characterized by menstrual irregularities, hyperandrogenism, and polycystic ovarian morphology [18]. The condition is associated with important reproductive and metabolic consequences, including infertility, insulin resistance, and an increased risk of type 2 diabetes mellitus [19,20].

Despite the growing burden of childhood obesity in India, data on the prevalence and patterns of dyslipidemia and its associated cardiometabolic comorbidities among children with overweight and obesity remain limited. Furthermore, information regarding the coexistence of hypertension, MASLD, and PCOS in this population is scarce. Therefore, the present study was undertaken to determine the prevalence and patterns of dyslipidemia among children with overweight and obesity attending a tertiary care center and to evaluate the prevalence of selected obesity-related comorbidities, including hypertension, MASLD, and PCOS.

## Materials and methods

This prospective observational study was conducted in the Department of Pediatrics at Apollo Hospitals, Chennai, India, over a one-year period from April 2018 to May 2019. Children aged 5-16 years who were classified as overweight or obese according to the Indian Academy of Pediatrics (IAP) body mass index (BMI) growth charts [21] were eligible for inclusion. Participants were recruited from the pediatric outpatient department during the study period. Only children whose parents or legal guardians provided written informed consent (and assent, where applicable, in accordance with institutional ethical guidelines) were enrolled. Children with endocrine disorders, syndromic obesity, chronic systemic illnesses affecting growth or metabolism, or those receiving medications known to influence body weight or lipid metabolism were excluded. Children whose parents or legal guardians declined to provide informed consent were also excluded.

The sample size was estimated based on an expected dyslipidemia prevalence of 48.8% reported by Bibiloni et al. [22], assuming a 95% confidence level and an allowable error of 10%. The minimum required sample size was calculated to be 96 participants. To account for possible attrition, a target sample size of 100 children was planned.

The primary aim of this study was to evaluate the lipid profile of children with overweight and obesity aged 5-16 years attending the pediatric outpatient department of a tertiary care hospital in Chennai, South India. Specifically, the study sought to determine the prevalence and patterns of dyslipidemia based on the measured lipid parameters, including TC, LDL-C, HDL-C, non-HDL-C, and TG. In addition, the study assessed the prevalence of selected obesity-related comorbidities, including hypertension, MASLD, and PMOS among postmenarchal girls, and examined their association with obesity and dyslipidemia.

A total of 135 children were screened for eligibility. Of these, 30 were excluded based on the predefined exclusion criteria, and five declined to provide informed consent. Consequently, 100 participants were enrolled in the study.

Demographic characteristics, birth history, and family history of diabetes mellitus and hypertension were recorded using a structured data collection form. All participants underwent a comprehensive clinical examination for markers of metabolic dysfunction, including acanthosis nigricans and acne, anthropometric measurements, blood pressure assessment, laboratory investigations, and abdominal ultrasonography.

Body weight was measured using a calibrated weighing scale, and height was measured using a stadiometer. BMI was calculated as weight (kg)/height (m²). Participants were categorized as overweight or obese according to the age- and sex-specific recommendations of the Indian Academy of Pediatrics BMI growth charts [21].

Blood pressure was measured using an automated oscillometric blood pressure monitor (Omron HEM-7270; Omron Healthcare Co., Ltd., Kyoto, Japan) with an appropriately sized cuff. After the participant had rested in a seated position for at least five minutes, three blood pressure measurements were obtained at 1-2-minute intervals. The average of the three readings was used for statistical analysis. Blood pressure measurements were interpreted according to the 2017 American Academy of Pediatrics (AAP) blood pressure percentile charts [23]. Children with blood pressure values at or above the 95th percentile for age, sex, and height were classified as hypertensive.

Following an overnight fast, venous blood samples were collected for lipid profile analysis. Serum lipid parameters and liver enzyme levels were analyzed using the Roche Cobas c311 automated chemistry analyzer (Roche Diagnostics, Mannheim, Germany) with the appropriate reagent kits. The lipid profile included TC, LDL-C, HDL-C, and TG. Non-HDL-C was calculated by subtracting HDL-C from the total cholesterol concentration. Non-HDL-C was included because it reflects the total burden of atherogenic lipoproteins and has been shown to predict future cardiovascular risk.

Dyslipidemia was defined according to the National Heart, Lung, and Blood Institute (NHLBI) recommendations [11] as the presence of one or more of the following abnormalities: TC ≥ 200 mg/dL, LDL-C ≥ 130 mg/dL, HDL-C ≤ 40 mg/dL, non-HDL-C ≥ 145 mg/dL, TG ≥ 100 mg/dL for children aged 0-9 years, or TG ≥ 130 mg/dL for those aged 10-19 years.

Participants were also evaluated for clinical markers of metabolic dysfunction, including acanthosis nigricans and acne. Menarchal status was determined based on parent or guardian report obtained during the structured medical history. Postmenarchal girls were further assessed for menstrual irregularities and clinical features suggestive of hyperandrogenism. They underwent evaluation for PMOS according to the Rotterdam diagnostic criteria [18], requiring the presence of at least two of the following three features: oligo- or anovulation, clinical or biochemical hyperandrogenism, and polycystic ovarian morphology on ultrasonography (≥12 follicles measuring 2-9 mm in diameter and/or an ovarian volume >10 cm³).

Abdominal and pelvic ultrasonography was performed in all participants by a single experienced radiologist who was blinded to the participants' clinical and laboratory findings. MASLD was graded according to the degree of hepatic steatosis identified on ultrasonography as Grade I (mild), Grade II (moderate), or Grade III (severe), based on standard ultrasonographic criteria.

The primary outcome of the study was the prevalence of dyslipidemia among children with overweight and obesity. Secondary outcomes included characterization of the patterns of lipid abnormalities, determination of the prevalence of hypertension, MASLD, and PMOS, and evaluation of the associations between dyslipidemia and selected demographic and clinical variables.

Data were entered into Microsoft Excel (Microsoft Corp., Redmond, WA, USA) and analyzed using IBM SPSS Statistics for Windows, Version 25.0 (Released 2017; IBM Corp., Armonk, NY, USA). Continuous variables were expressed as mean ± standard deviation (SD), whereas categorical variables were presented as frequencies and percentages. Comparisons between categorical variables were performed using the chi-square test or Fisher's exact test, as appropriate. Continuous variables were compared using the independent-samples t-test. A two-sided p-value <0.05 was considered statistically significant.

## Results

A total of 100 children with overweight and obesity aged 5-16 years were included in the study during the study period. The mean age of the study population was 11.6 ± 2.56 years. Females constituted 53 (53%) of the participants, while males accounted for 47 (47%). Most participants belonged to the early adolescent age group (10-13 years), representing 55% (55/100) of the study population. Table 1 summarizes the baseline demographic and clinical characteristics of the study population. 

**Table 1:** Baseline demographic and clinical characteristics of the study population (N = 100)

Characteristic	n (%)
Age group	
5-9 years	20 (20)
10-13 years	55 (55)
14-16 years	25 (25)
Sex	
Male	47 (47)
Female	53 (53)
Birth weight for gestational age	
Appropriate for gestational age	88 (88)
Small for gestational age	7 (7)
Large for gestational age	5 (5)
Family history of diabetes mellitus	42 (42)
Family history of hypertension	25 (25)
BMI category	
Overweight	80 (80)
Obese	20 (20)
Clinical findings	
Acne	40 (40)
Acanthosis nigricans	34 (34)

The overall prevalence of dyslipidemia was 84% (84/100; 95% confidence interval (CI): 75.6%-89.9%). Low HDL-C was the most frequently observed lipid abnormality, followed by elevated triglycerides. Elevated LDL-C was the least common lipid abnormality. Table 2 summarizes the distribution of lipid abnormalities.

**Table 2:** Distribution of lipid abnormalities among study participants (N = 100) HDL-C: high-density lipoprotein cholesterol, LDL-C: low-density lipoprotein cholesterol.

Lipid parameter	Normal, n (%)	Abnormal, n (%)
Total cholesterol	92 (92)	8 (8)
LDL-C	98 (98)	2 (2)
HDL-C	43 (43)	57 (57)
Non-HDL-C	86 (86)	14 (14)
Triglycerides	56 (56)	44 (44)

Analysis of obesity-related comorbidities demonstrated that MASLD was present in 32 participants (32%; 95% CI: 23.7%-41.7%), and hypertension was identified in 7 participants (7%; 95% CI: 3.4%-13.7%). Among the 15 postmenarchal girls screened, PMOS was detected in 4 (26.7%; 95% CI: 10.9%-52.0%). Table 3 summarizes the prevalence of major obesity-associated comorbidities.

**Table 3:** Prevalence of obesity-associated comorbidities MASLD: metabolic dysfunction-associated steatotic liver disease, PMOS: polyendocrine metabolic ovarian syndrome.

Comorbidity	n (%)	95% CI
Dyslipidemia	84/100 (84.0%)	75.6%-89.9%
MASLD	32/100 (32.0%)	23.7%-41.7%
Hypertension	7/100 (7.0%)	3.4%-13.7%
PMOS	4/15 (26.7%)	10.9%-52.0%

Among the 32 participants with fatty liver disease, Grade I steatosis was the predominant ultrasonographic finding. No participant demonstrated Grade III steatosis, as shown in Table 4.

**Table 4:** Severity of MASLD among affected participants MASLD: metabolic dysfunction-associated steatotic liver disease.

Grade of hepatic steatosis	n (%)
Grade I	24 (75.0)
Grade II	8 (25.0)
Grade III	0 (0)
Total	32 (100)

Female participants demonstrated a significantly higher prevalence of dyslipidemia than male participants (94.3% vs. 72.3%, p = 0.003, Pearson χ² = 8.97, df = 1). Hypertension was significantly more common among children with obesity than among children with overweight (20.0% vs. 3.8%, p = 0.028, Fisher's exact test; OR = 6.42, 95% CI: 1.31-31.49). No significant association was observed between dyslipidemia and birth weight category, family history of diabetes mellitus, family history of hypertension, or hypertension status. MASLD was not significantly associated with dyslipidemia. However, elevated liver enzyme levels, including serum glutamic-pyruvic transaminase (SGPT) and gamma-glutamyl transferase (GGT), demonstrated a significant association with the severity of hepatic steatosis (p < 0.001) (Table 5).

**Table 5:** Significant associations observed in the study Values for MASLD severity rows are expressed as mean ± standard deviation. MASLD: metabolic dysfunction-associated steatotic liver disease, SGOT: serum glutamic-oxaloacetic transaminase, SGPT: serum glutamic-pyruvic transaminase, GGT: gamma-glutamyl transferase.

Variable	Comparison	Group	Value	Statistical test	p-value
Sex	Dyslipidemia	Female	50/53 (94.3%)	Pearson chi-square, χ² = 8.97	0.003
		Male	34/47 (72.3%)		
BMI category	Hypertension	Obese	4/20 (20.0%)	Fisher's exact test, OR = 6.42	0.028
		Overweight	3/80 (3.8%)		
MASLD severity	SGOT	Grade I	28.17 ± 10.65	One-way ANOVA	0.006
		Grade II	44.13 ± 22.47		
		No MASLD	29.65 ± 11.46		
MASLD severity	SGPT	Grade I	32.67 ± 14.68	One-way ANOVA	<0.001
		Grade II	73.13 ± 46.52		
		No MASLD	29.19 ± 11.46		
MASLD severity	GGT	Grade I	21.00 ± 7.87	One-way ANOVA	<0.001
		Grade II	42.63 ± 38.52		
		No MASLD	19.21 ± 8.70		

## Discussion

This prospective observational study evaluated the prevalence and pattern of dyslipidemia and associated obesity-related comorbidities among children with overweight and obesity attending a tertiary care center in South India. The principal findings included a high prevalence of dyslipidemia (84%), a predominance of low HDL-C and hypertriglyceridemia, a substantial burden of MASLD (32%), and the presence of hypertension in 7% of participants. In addition, more than one-fourth of the postmenarchal girls fulfilled the diagnostic criteria for PMOS. These findings underscore the substantial cardiometabolic burden associated with childhood overweight and obesity and highlight the importance of early screening in high-risk pediatric populations.

Demographic characteristics

The mean age of the participants in the present study was 11.6 ± 2.56 years, with the majority belonging to the early adolescent age group. A slight female predominance was observed. Although birth weight was not significantly associated with obesity severity or dyslipidemia, all children born large for gestational age had either overweight or obesity. Similar observations were reported by Qiao et al., who demonstrated an association between higher birth weight and an increased risk of childhood obesity across multiple countries [24], suggesting that fetal and early-life growth patterns may influence later adiposity.

Prevalence of dyslipidemia

The most important finding of this study was the exceptionally high prevalence of dyslipidemia, which was identified in 84% of children with overweight and obesity. This prevalence was higher than that reported in several Indian studies but remained broadly consistent with the established relationship between obesity and metabolic dysfunction. Minakshi and Chithambaram [25] reported dyslipidemia in 69% of Indian children with overweight and obesity, while Nijaguna [26] documented a prevalence of approximately 63%. These differences in prevalence rates may be explained by variations in study populations, diagnostic criteria, age distribution, and regional dietary practices.

The high prevalence observed in the present study reinforces the concept that dyslipidemia is one of the earliest metabolic consequences of childhood obesity. Importantly, dyslipidemia was present not only among children with obesity but also among children with overweight, suggesting that metabolic abnormalities may develop before severe obesity becomes established. This finding supports recommendations advocating metabolic evaluation even in children classified as having overweight rather than obesity [11].

Pattern of lipid abnormalities

Low HDL-C was the most common lipid abnormality, affecting 57% of participants, followed by elevated triglycerides, which were observed in 44% of the study population. Elevated TC, elevated non-HDL-C, and elevated LDL-C were considerably less common.

This pattern is consistent with the characteristic dyslipidemia frequently observed in obesity and insulin resistance. Low HDL-C and hypertriglyceridemia represent the key components of atherogenic dyslipidemia, a lipid profile that is particularly prevalent among South Asian populations and is associated with an increased cardiovascular risk [27]. Similar findings have been reported in the National Health and Nutrition Examination Survey (NHANES) and in previous Indian studies, including reports from Karnataka and rural Jaipur, where low HDL-C was identified as the predominant lipid abnormality among children with overweight and obesity [25,26,28].

The predominance of low HDL-C has important clinical implications because HDL-C plays a protective role in reverse cholesterol transport and vascular health. Reduced HDL-C levels during childhood may contribute to the early development of atherosclerotic changes and increase the long-term risk of cardiovascular disease [2,10].

Sex differences in dyslipidemia

A significant association (p < 0.05) was observed between female sex and dyslipidemia. Nearly all female participants demonstrated at least one lipid abnormality, and the prevalence was significantly higher than that observed among male participants.

The reasons for this sex-related difference are likely multifactorial and may involve hormonal influences, body fat distribution, pubertal changes, dietary patterns, and physical activity levels. Pubertal maturation, in particular, is known to influence lipid metabolism, with estrogen exposure in girls and rising testosterone levels in boys producing divergent effects on HDL-C and triglyceride concentrations as puberty progresses. Because girls generally enter puberty earlier than boys of the same chronological age, differences in pubertal stage may plausibly have contributed to the observed sex difference. However, pubertal staging (e.g., Tanner staging) was not formally assessed in this study; therefore, its independent contribution to the observed sex difference in dyslipidemia could not be evaluated. Additionally, because of the relatively small sample size, these findings should be interpreted with caution and require confirmation in larger studies with participants matched for pubertal stage.

MSALD

MASLD was identified in nearly one-third of the participants, highlighting the close relationship between obesity and hepatic steatosis in children. Most affected children demonstrated Grade I steatosis, while no participant exhibited Grade III steatosis.

Although MASLD was more common among children with dyslipidemia, a statistically significant association was not demonstrated. Nevertheless, a strong association was observed between elevated liver enzyme levels and the severity of hepatic steatosis. This finding is clinically relevant because liver enzyme abnormalities may serve as accessible markers for identifying children who require further evaluation for MASLD.

The prevalence observed in the present study is consistent with previous reports that have identified MASLD as one of the most common obesity-related complications in children [15,16]. Given the potential progression from simple steatosis to steatohepatitis, fibrosis, and cirrhosis, early detection and lifestyle modification remain essential [17]. Previous longitudinal studies have demonstrated the progressive nature of pediatric MASLD and its potential for long-term hepatic complications [15-17].

Hypertension

Hypertension was present in 7% of the participants. Although this prevalence is lower than that reported by Yadav et al. [29], it remains clinically important because elevated blood pressure during childhood is known to track into adulthood and contribute to future cardiovascular disease.

A particularly important finding was the significantly higher prevalence of hypertension among children with obesity than among children with overweight. This observation supports previous studies demonstrating a positive relationship between increasing adiposity, elevated blood pressure, and other cardiovascular risk factors among children with obesity [13]. Potential mechanisms include sympathetic nervous system activation, insulin resistance, endothelial dysfunction, and alterations in renal sodium handling. Routine blood pressure monitoring should therefore form an integral component of obesity assessment in pediatric practice, particularly among children with severe obesity [23].

Polyendocrine metabolic ovarian syndrome

Among the postmenarchal girls, the prevalence of PMOS was 26.7% in our study. This finding is comparable to previously reported rates by Ramanand et al. [30] among adolescent girls with overweight and obesity in India. All affected participants reported menstrual irregularities, and most exhibited clinical features suggestive of hyperandrogenism or insulin resistance.

The association between obesity and PMOS is well established. Childhood obesity contributes significantly to the development of adolescent PMOS and is frequently associated with adverse lipid profiles and insulin resistance [19,20]. Excess adiposity contributes to insulin resistance and hyperinsulinemia, which, in turn, promote ovarian androgen production and reproductive dysfunction [20]. Early recognition of PMOS is important because the condition is associated with infertility, metabolic syndrome, type 2 diabetes mellitus, and adverse cardiovascular outcomes later in life [18].

Diagnosing PMOS during early adolescence poses particular challenges. Physiological anovulatory cycles are common during the first two to three years after menarche and can result in menstrual irregularities resembling the core diagnostic feature of PMOS, even in the absence of true ovarian dysfunction [18]. Clinical features such as acne and mild hirsutism also overlap considerably with normal pubertal changes, further complicating the diagnosis in this age group. For this reason, recent updates to international guidelines recommend caution when diagnosing PMOS during early adolescence, as physiological menstrual irregularity is common after menarche [31]. Current adolescent-specific guidelines also recommend requiring both hyperandrogenism and ovulatory dysfunction for diagnosis in this age group, without relying on ovarian ultrasonographic morphology, which is a criterion validated for adult women and is not recommended for use in adolescents [31]. In this study, menarchal status was ascertained by parental report rather than by direct clinical or biochemical verification, and PMOS was diagnosed using the adult Rotterdam criteria (including ultrasonographic assessment of ovarian morphology) at a single time point. These factors, together with the small number of postmenarchal girls evaluated (n = 15), warrant caution when distinguishing physiological from pathological menstrual irregularity, may have led to an overestimation of the true prevalence of PMOS in this subgroup, and support the need for longitudinal follow-up using adolescent-specific criteria to confirm the diagnosis in affected girls.

Clinical implications

The findings of this study have important implications for pediatric practice. The high prevalence of dyslipidemia and associated metabolic abnormalities observed even among children with overweight suggests that BMI, while a practical and widely used screening tool for adiposity, does not fully capture the cardiometabolic risk present in this population. Comprehensive metabolic evaluation, including lipid screening and blood pressure assessment, should therefore be considered for children with overweight and obesity, particularly during adolescence, rather than relying on BMI-based screening alone [11,23].

Early identification of dyslipidemia provides an opportunity for intervention before the development of irreversible cardiovascular disease. Lifestyle modification remains the cornerstone of management and includes dietary optimization, increased physical activity, weight reduction, and reduction of sedentary behaviors. In selected cases, pharmacologic therapy may be considered according to established pediatric guidelines [11].

Future directions

Large multicenter studies are required to determine the true prevalence of dyslipidemia and obesity-related comorbidities among Indian children. Longitudinal studies would also help clarify the progression of dyslipidemia, hypertension, MASLD, and PMOS into adulthood. The development of population-specific screening recommendations may further improve the early identification and management of high-risk children.

Strengths and limitations

This study has several strengths, including its prospective design, comprehensive clinical and biochemical assessment of children with overweight and obesity, and the use of standardized diagnostic definitions for dyslipidemia, hypertension, and MASLD. The findings contribute valuable data from an Indian pediatric population, for which such data remain relatively limited.

This study also has several limitations. First, the sample size was relatively small and included only 100 participants, limiting the statistical power and generalizability of the findings. Second, the cross-sectional design does not permit causal inference regarding the observed associations; therefore, all reported relationships should be interpreted as associative rather than causal. Third, PMOS was diagnosed using the Rotterdam criteria (including ultrasonographic assessment of ovarian morphology), which were developed and validated in adult women. Current international evidence-based guidelines for adolescents instead recommend requiring both hyperandrogenism and ovulatory dysfunction, without reliance on ovarian ultrasonographic morphology, given the physiological overlap between normal pubertal changes and features of PMOS in this age group. Hyperandrogenism in our cohort was also assessed clinically, without the use of a standardized scoring tool, such as the Ferriman-Gallwey score, or a biochemical androgen panel (e.g., total or free testosterone and DHEAS). Applying adult-derived diagnostic criteria in this manner may have overestimated the true prevalence of PMOS in this adolescent subgroup, and this should be considered when interpreting our findings. Fourth, fasting plasma glucose, glycated hemoglobin (HbA1c), and fasting insulin levels were not measured, and insulin resistance could not be quantified using indices such as the homeostatic model assessment of insulin resistance (HOMA-IR). Given that acanthosis nigricans, a clinical marker of insulin resistance, was present in 34% of participants, the absence of glycemic and insulin data leaves an important gap in characterizing the metabolic risk observed in this cohort. Future studies should incorporate these parameters to better delineate the relationship between adiposity, insulin resistance, and the comorbidities described here. Fifth, pubertal staging (e.g., Tanner staging) was not formally assessed. Pubertal maturation is known to influence lipid metabolism, and although the sexes did not differ appreciably in age distribution, residual confounding by pubertal status cannot be excluded, particularly with respect to the sex differences in dyslipidemia observed in this study. Similarly, factors such as body fat distribution were not assessed and may have contributed to dyslipidemia but were not evaluated in the present study. Sixth, statistical comparisons in this study were bivariate, and associations such as the sex difference in dyslipidemia were not adjusted for potential confounders, including age and pubertal status, using multivariable methods. Therefore, the reported associations should be interpreted as unadjusted. Finally, because the study population consisted exclusively of children with overweight and obesity, comparisons with normal-weight controls were not possible.

## Conclusions

This prospective observational study demonstrated a high prevalence of dyslipidemia (84%) among children with overweight and obesity attending a tertiary care center in South India, with low HDL-C and hypertriglyceridemia constituting the predominant lipid pattern. A substantial proportion of participants also had MASLD (32%), and hypertension was significantly more common among children with obesity than among children with overweight. Among the postmenarchal girls, more than one-fourth met the diagnostic criteria for PMOS, although this estimate should be interpreted with caution given the small subgroup size and the use of adult-derived diagnostic criteria.

These findings underscore the substantial cardiometabolic burden associated with childhood overweight and obesity and highlight the importance of early, comprehensive metabolic screening beyond BMI alone. Given the modest sample size, single-center design, and the absence of glycemic and pubertal staging data, larger multicenter studies incorporating these parameters are needed to confirm these findings and inform population-specific screening strategies for high-risk children.

## References

[1] Kimm SY, Obarzanek E (2002). Childhood obesity: a new pandemic of the new millennium. Pediatrics.

[2] Berenson GS, Srinivasan SR, Bao W, Newman WP 3rd, Tracy RE, Wattigney WA (1998). Association between multiple cardiovascular risk factors and atherosclerosis in children and young adults. N Engl J Med.

[3] Lauer RM, Lee J, Clarke WR (1988). Factors affecting the relationship between childhood and adult cholesterol levels: the Muscatine Study. Pediatrics.

[4] Singh AS, Mulder C, Twisk JW, van Mechelen W, Chinapaw MJ (2008). Tracking of childhood overweight into adulthood: a systematic review of the literature. Obes Rev.

[5] Chudasama RK, Eshwar TKM, Eshwar ST, Thakkar D (2016). Prevalence of obesity and overweight among school children aged 8-18 years in Rajkot, Gujarat. Indian Pediatr.

[6] Ranjani H, Mehreen TS, Pradeepa R, Anjana RM, Garg R, Anand K, Mohan V (2016). Epidemiology of childhood overweight & obesity in India: a systematic review. Indian J Med Res.

[7] Herrington L, Susi A, Gorman G, Nylund CM, Hisle-Gorman E (2019). Factors affecting pediatric dyslipidemia screening and treatment. Clin Pediatr (Phila).

[8] Kit BK, Kuklina E, Carroll MD, Ostchega Y, Freedman DS, Ogden CL (2015). Prevalence of and trends in dyslipidemia and blood pressure among US children and adolescents, 1999-2012. JAMA Pediatr.

[9] Williams CL, Hayman LL, Daniels SR, Robinson TN, Steinberger J, Paridon S, Bazzarre T (2002). Cardiovascular health in childhood: a statement for health professionals from the Committee on Atherosclerosis, Hypertension, and Obesity in the Young (AHOY) of the Council on Cardiovascular Disease in the Young, American Heart Association. Circulation.

[10] Srinivasan SR, Frontini MG, Xu J, Berenson GS (2006). Utility of childhood non-high-density lipoprotein cholesterol levels in predicting adult dyslipidemia and other cardiovascular risks: the Bogalusa Heart Study. Pediatrics.

[11] Expert Panel on Integrated Guidelines for Cardiovascular Health and Risk Reduction in Children and Adolescents (2011). Expert panel on integrated guidelines for cardiovascular health and risk reduction in children and adolescents: summary report. Pediatrics.

[12] McCrindle BW (2018). Pathogenesis and management of dyslipidemia in obese children. Pediatric Obesity. Contemporary Endocrinology.

[13] Chandrasekhar T, Suchitra MM, Pallavi M, L N Srinivasa Rao PV, Sachan A (2017). Risk factors for cardiovascular disease in obese children. Indian Pediatr.

[14] Brady TM (2017). Obesity-related hypertension in children. Front Pediatr.

[15] Dowla S, Aslibekyan S, Goss A, Fontaine K, Ashraf AP (2018). Dyslipidemia is associated with pediatric nonalcoholic fatty liver disease. J Clin Lipidol.

[16] Schwimmer JB, Deutsch R, Kahen T, Lavine JE, Stanley C, Behling C (2006). Prevalence of fatty liver in children and adolescents. Pediatrics.

[17] Feldstein AE, Charatcharoenwitthaya P, Treeprasertsuk S, Benson JT, Enders FB, Angulo P (2009). The natural history of nonalcoholic fatty liver disease in children. Gut.

[18] The Rotterdam ESHRE/ASRM-Sponsored PCOS Consensus Workshop Group (2004). Revised 2003 consensus on diagnostic criteria and long-term health risks related to polycystic ovary syndrome. Fertil Steril.

[19] Anderson AD, Solorzano CM, McCartney CR (2014). Childhood obesity and its impact on the development of adolescent PCOS. Semin Reprod Med.

[20] Wild RA, Rizzo M, Clifton S, Carmina E (2011). Lipid levels in polycystic ovary syndrome: systematic review and meta-analysis. Fertil Steril.

[21] Khadilkar V, Yadav S, Agrawal KK (2015). Revised IAP growth charts for height, weight and body mass index for 5- to 18-year-old Indian children. Indian Pediatr.

[22] Bibiloni MM, Salas R, Pons A, Tur JA (2015). Prevalence of dyslipidaemia and associated risk factors among Balearic Islands adolescents, a Mediterranean region. Eur J Clin Nutr.

[23] Flynn JT, Kaelber DC, Baker-Smith CM (2017). Clinical practice guideline for screening and management of high blood pressure in children and adolescents. Pediatrics.

[24] Qiao Y, Ma J, Wang Y (2015). Birth weight and childhood obesity: a 12-country study. Int J Obes Suppl.

[25] Minakshi B, Chithambaram NS (2016). Abnormalities of lipid profile in overweight and obese Indian children. Int J Pediatr Res.

[26] Nijaguna N, Vani HN, Niranjan HS, Suresh TN, Sanjeeva GN (2015). Study of lipid profile and prevalence of dyslipidemia in adolescent school children from Karnataka. Int J Pharm Biol Sci.

[27] Chandra KS, Bansal M, Nair T (2014). Consensus statement on management of dyslipidemia in Indian subjects. Indian Heart J.

[28] Duncan GE, Li SM, Zhou XH (2004). Prevalence and trends of a metabolic syndrome phenotype among U.S. adolescents, 1999-2000. Diabetes Care.

[29] Yadav PKS, Yadav MB, Yadav C (2019). Prevalence of overweight, obesity and hypertension among school-going children in Uttar Pradesh, India. Int J Contemp Pediatr.

[30] Ramanand SJ, Ghongane BB, Ramanand JB, Patwardhan MH, Ghanghas RR, Jain SS (2013). Clinical characteristics of polycystic ovary syndrome in Indian women. Indian J Endocrinol Metab.

[31] Teede HJ, Tay CT, Laven JJ (2023). Recommendations from the 2023 international evidence-based guideline for the assessment and management of polycystic ovary syndrome. Eur J Endocrinol.

